# Individual recognition of Eurasian beavers (*Castor fiber*) by their tail patterns using a computer‐assisted pattern‐identification algorithm

**DOI:** 10.1002/ece3.10922

**Published:** 2024-02-13

**Authors:** Margarete Dytkowicz, Marcello Tania, Rachel Hinds, William M. Megill, Tillmann K. Buttschardt, Frank Rosell

**Affiliations:** ^1^ FabLab Blue, Faculty of Technology and Bionics University of Applied Sciences Kleve Germany; ^2^ Research Group Applied Landscape Ecology and Ecological Planning, Institute of Landscape Ecology WWU Münster Münster Germany; ^3^ Department of Natural Sciences and Environmental Health, Faculty of Technology, Natural Sciences and Maritime Sciences University of South‐Eastern Norway Bø i Telemark Norway

**Keywords:** *Castor fiber*, Eurasian beaver, individual pattern recognition, non‐invasive mark–recapture, scale‐invariant feature transform, wildlife ecology

## Abstract

Individual recognition of animals is an important aspect of ecological sciences. Photograph‐based individual recognition options are of particular importance since these represent a non‐invasive method to distinguish and identify individual animals. Recent developments and improvements in computer‐based approaches make possible a faster semi‐automated evaluation of large image databases than was previously possible. We tested the Scale Invariant Feature Transform (SIFT) algorithm, which extracts distinctive invariant features of images robust to illumination, rotation or scaling of images. We applied this algorithm to a dataset of 800 tail pattern images from 100 individual Eurasian beavers (*Castor fiber*) collected as part of the Norwegian Beaver Project (NBP). Images were taken using a single‐lens reflex camera and the pattern of scales on the tail, similar to a human fingerprint, was extracted using freely accessible image processing programs. The focus for individual recognition was not on the shape or the scarring of the tail, but purely on the individual scale pattern on the upper (dorsal) surface of the tail. The images were taken from two different heights above ground, and the largest possible area of the tail was extracted. The available data set was split in a ratio of 80% for training and 20% for testing. Overall, our study achieved an accuracy of 95.7%. We show that it is possible to distinguish individual beavers from their tail scale pattern images using the SIFT algorithm.

## Graphical abstract

The individual differentiation of beavers based on images and their scale pattern is a non‐invasive method. The evaluation of the images using a SIFT algorithm enables an exact differentiation of the animals. Thus, this approach represents a new non‐invasive method for beaver monitoring.

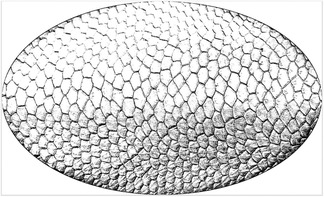

## 1 INTRODUCTION

Individual recognition of animals make it possible to obtain detailed information and make statements about population size and distribution and is therefore an important aspect of ecological sciences (Deutsch, 2015; Kelly, 2001; Lahiri et al., 2011; Ravela & Gamble, 2004). In particular, non‐invasive methods based on photo‐identification are increasingly coming into focus (Association for the Study of Animal Behaviour/Animal Society Behaviour (ASAB/ASB), 2012; Schofield et al., 2020). This is possible because many animals show naturally occurring characteristics that can be used for individual identification (Ardovini et al., 2007; Kelly, 2001; Urian et al., 2015). The ease of use and reliability of modern digital cameras often result in huge numbers of images. Even if it is possible to evaluate the data records manually, it may be prohibitively time‐consuming (Bradfield, 2004; Sarmento et al., 2009; Schofield et al., 2020; Wells & Scott, 1990; Wölfl, 2008). Computer‐based methods have therefore been used to facilitate the evaluation of the data sets (Falzon et al., 2019; Ferreira et al., 2020; Hillman et al., 2003; Lahiri et al., 2011). Some methods are based on 3D‐computer matching systems, while others use different types of 2D pattern‐matching algorithms (Hiby et al., 2009; Kelly, 2001; Swanson et al., 2015).

Three such programs are I^3^S, HotSpotter and Wild‐ID, which are capable of processing a large amount of image material within reasonable time and budget constraints (Nipko et al., 2020; Speed et al., 2007).

Recent advances in deep learning, particularly on convolutional neural networks (CNN) and deep convolutional neural networks (DCNN), are showing great potential for application on photo‐identification. CNNs and DCNNs have been used to identify individual birds from images and to automatically count and differentiate distinct animal species (Ferreira et al., 2020; Norouzzadeh et al., 2018). Such computer‐based approaches even showed adequate results in the individual recognition of mammalian species that lack unique markings, in particular for facial recognition of western lowland gorillas (*Gorilla gorilla gorilla*) (Brust et al., 2017), brown bears (*Ursus arctos*) (Clapham et al., 2020), several endangered primates species (Deb et al., 2018) and chimpanzees (*Pan troglodytes*) (Schofield et al., 2019). A large number of algorithms are available to the user for using CNNs or DCNNs. These algorithms can however be difficult to apply, because they are sensitive to variation in image quality, lighting conditions, shading, reflections, the angle of the shot, the framing of or distance to the subjects and even the type of pigmentation type to be used for identification.

Another approach to computer‐based individual identification of animals is the Scale Invariant Feature Transform (SIFT) algorithm. The SIFT algorithm is in particular robust to noise, illumination, rotation or scaling of the images (Bolger et al., 2012; Brust et al., 2017; Huang et al., 2008). SIFT is an image descriptor for image‐based matching and recognition (Lindeberg, 2012; Lowe, 1999, 2004a). This approach enables the extraction of distinctive invariant features from images. It transforms an input image into a collection of local features (Alhwarin et al., 2008).

The algorithm makes comparisons between the images used for the greatest possible proportions of the same features. The SIFT algorithm has been used successfully with photographs to individually identify African penguins (*Spheniscus demersus*) by their chest patterns (Bolger et al., 2012) and Masai giraffes (*Giraffa camelopardalis tippelskirchi*) by their fur pattern (Burghardt & Campbell, 2007). Successful results for the individual identification of two aquatic species of manta rays (*Manta alfredi* and *M. birostris*) using the SIFT algorithm could also be obtained (Town et al., 2013). The use of the SIFT algorithm on images of semi‐aquatic mammals for individual identification has to the best of our knowledge not yet been attempted.

It is particularly interesting to identify Eurasian beavers (*Castor fiber*) individually and thus to be able to make statements about their number and distribution as the animals recover from local extirpation in large parts of Europe (Nolet & Rosell, 1998) intensive protective measures and reintroduction projects have allowed the population to increase from c. 1200 to c. 1.5 million animals (Halley et al., 2021; Nolet & Rosell, 1998). Mark–recapture studies using individual beaver recognition have relied so far on PIT tags (Briggs et al., 2021; Mayer et al., 2022), GPS tags (Mayer et al., 2019), ear tags, modified VHF‐ear tags for the tail, as well as neck radio collars or backpack harnesses (Arjo et al., 2008) or even intraperitoneal radio transmitters (Ranheim et al., 2004). However, it has been shown that these mark–recapture actions can result in negative effects, such as loss of body mass or even death (Mortensen & Rosell, 2020; Ranheim et al., 2004; Robstad et al., 2021). Some animals exhibit natural tail wounds and scars, which can be used for individual identification, however not every animal shows such marks, and in particular young beavers do not generally exhibit wounds or scars (Mayer et al., 2019, 2020; Schwaiger & Schwemmer, 2012). However, the beaver tail displays other characteristics that make it an exciting approach to individual identification of the species. One study showed that the size of the tail does not increase for adult individuals of North American beavers (*C. canadensis*) (Smith & Jenkins, 1997). It seems reasonable to assume the same holds for Eurasian beaver. Additionally, in one study of individual identification of deceased beavers by their tail pattern using images taken with a single‐lens‐reflex camera (SLR), it was shown that a 100% accuracy of identification could be achieved by visual comparison (Hinds et al., 2023). In a different study based on visual comparison of images taken in the wild using camera traps to capture images in the wild, only 272 of 790 images (34%) could be identified (Schwaiger & Schwemmer, 2012) based on special features of the tail like scars and wounds. Neither of these studies identified individual beaver tails using any kind of computer algorithm.

In this study, we used the SIFT algorithm to individually recognise Eurasian beavers by the scale patterns on the dorsal side of their tails.

## 2 MATERIALS AND METHODS

We used a three‐step process similar toother computer‐based methods, which includes a database of recorded images of each individual beaver, a method for extracting the patterns of the images, and an algorithm for pattern‐matching which compares the pattern information of each new image to the existing images in the database (Bolger et al., 2012). It is known that increasing the number of individuals and images in the database and training should increase accuracy and limit overfitting (Brust et al., 2017; Clapham et al., 2020).

### 2.1 Data collection

**FIGURE 1:**
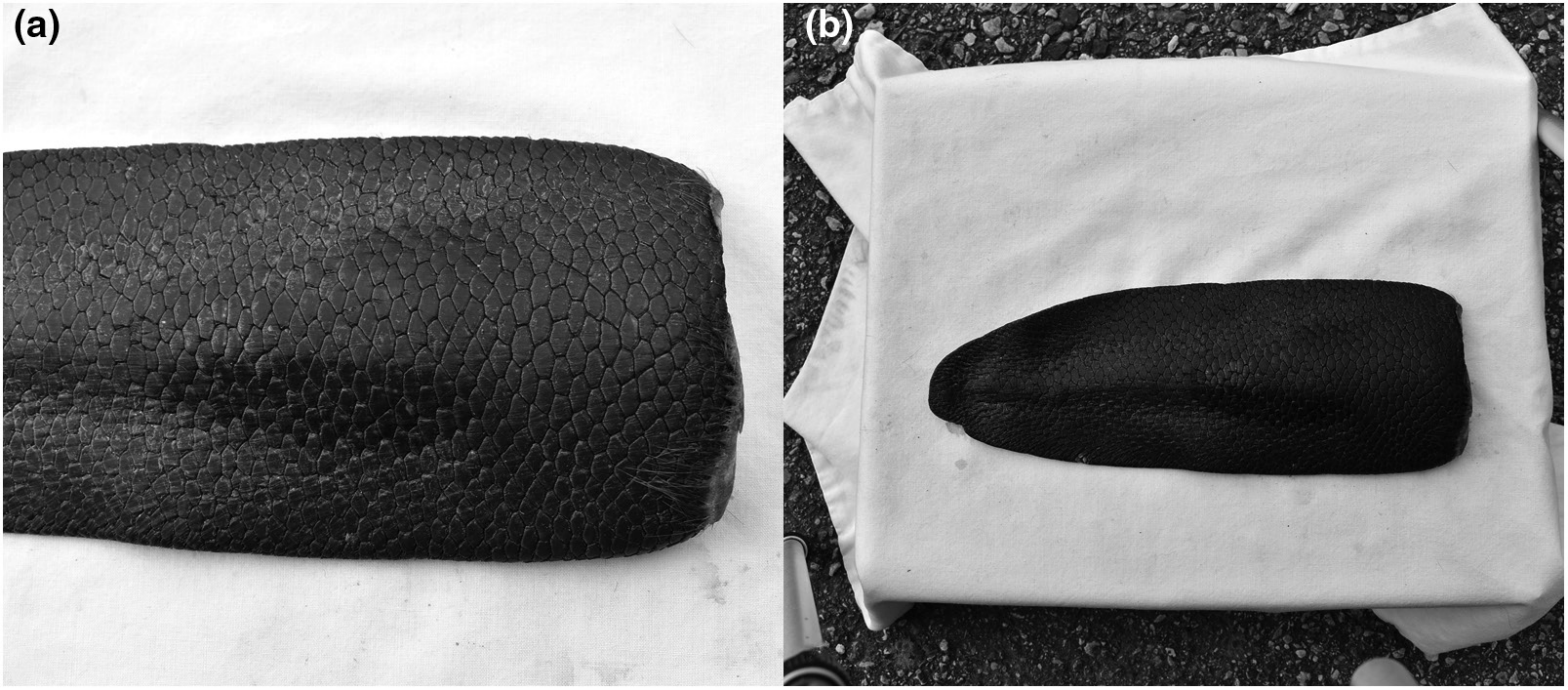
Grey‐scaled images of beaver tails from previously hunted beavers in Norway taken by a single‐lens reflex camera. (a) Image from the group ‘close’. (b) Image from the group ‘far’.

A total of 800 images of 100 individual hunted beavers in Norway were used. Images were taken with an SLR camera under good, mostly indoor, lighting conditions and showed the upper (dorsal) side of beaver tails on a table. Due to pandemic‐related restrictions on laboratory access, some of the images had to be taken outside, resulting in different lighting conditions. To facilitate comparison with a previous non‐computerised study (Hinds et al., 2023), images were taken from two different heights above the tails, chosen to standardise the size of the tail images. Images were split into two groups: ‘close’ (the length of the tail filled four grid squares on the camera viewfinder) and ‘far’ (two grid squares). Representative images are shown in Figure 1a,b. The different heights are used to compare accuracy at different levels; whether the recording height influences the accuracy or not.

Images of the category ‘close’ show the beaver tail in more detail than images of the category ‘far’, in which the beaver tail as a whole and objects from the environment (table legs and floor) can be seen. These environmental influences can negatively affect the quality of the image, in particular in our case this means that the scale pattern is no longer clearly displayed. The images of the ‘close’ group show better resolution than the ‘far’ group since the second group is more influenced by the incidence of light and shadow. Another difference between images from the two groups is that the images from the ‘far’ group show the beaver tail darker than images from the ‘close’ group because the exposure times of the two groups are different. Corrections were straightforward to do and are described in the next section.

Due to the amount of data, and consequently, to avoid overloading or crashing the computer, each of the two groups was divided into four samples of 25 animals each with four images for testing, resulting in 100 images per sample and 400 images for each of the two groups.

### 2.2 Pre‐processing of images

**FIGURE 2:**
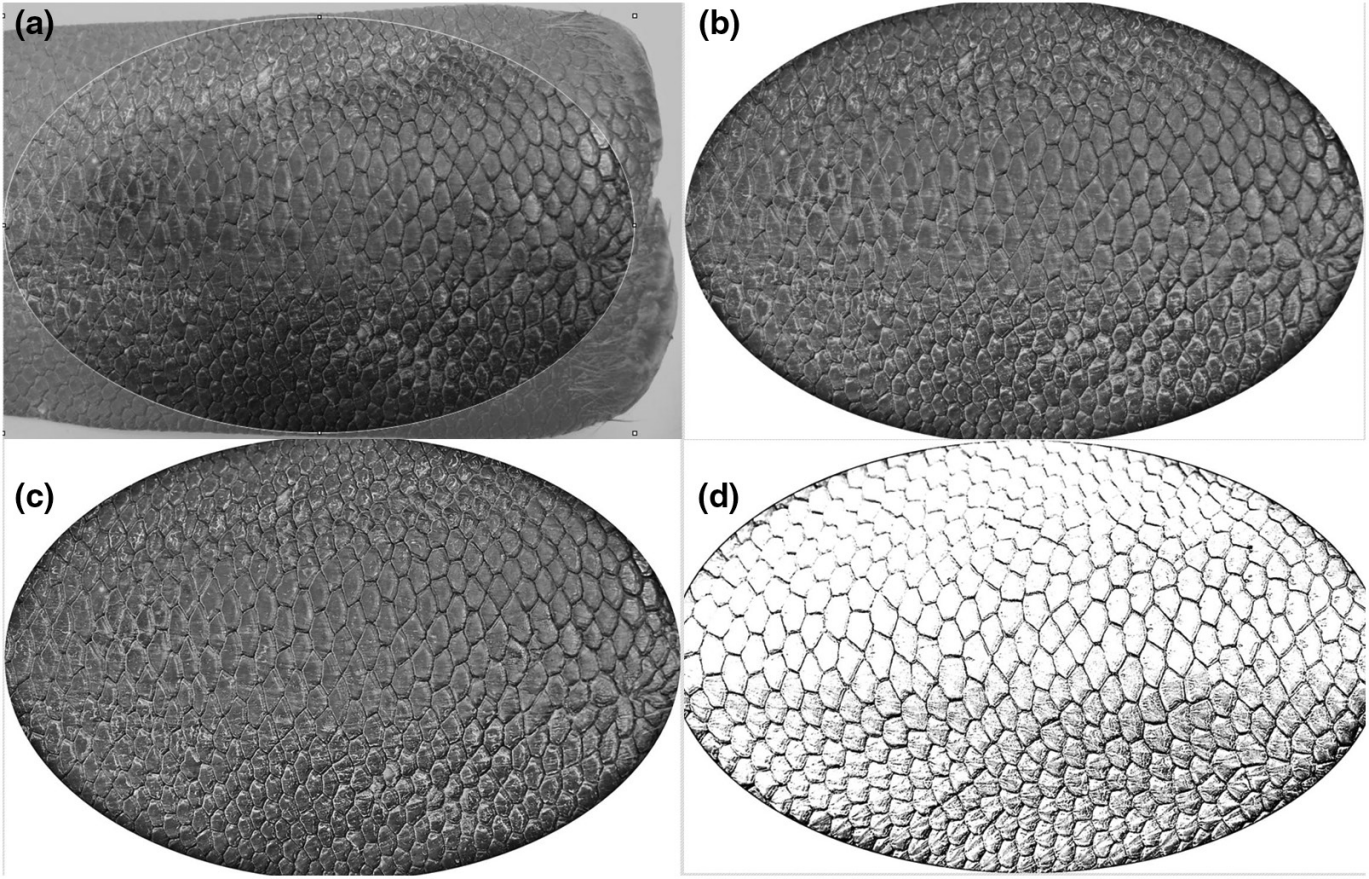
Overview of the editing process for images of the group ‘close’ using the image editing program ‘Photoscape’. (a) The cutting of the largest possible area of the beaver tail. (b) The use of the backlight function. (c) The sharpening adjustment. (d) The final processing to show the pattern more clearly. The areas ‘intensify’, ‘brighten’, as well as the exposure, the contrast, the brightness, as well as ‘gamma brightness’ are changed.

**FIGURE 3:**
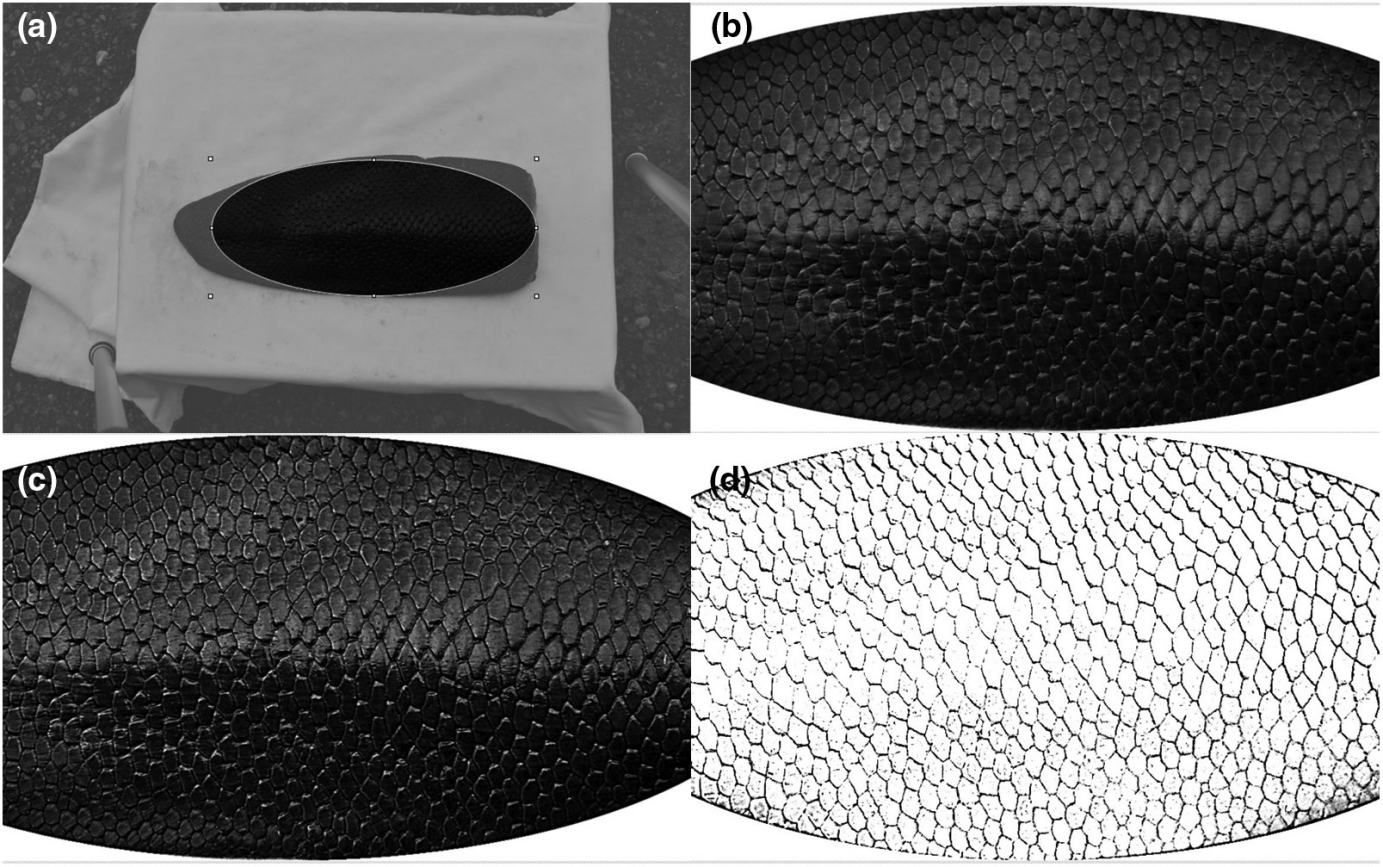
Overview of the editing process for images of the group ‘far’ using the image editing program ‘Photoscape’. (a) The cutting of the largest possible area of the beaver tail. (b) The use of the backlight function. (c) The sharpening adjustment. (d) The final processing to show the pattern more clearly. The areas ‘intensify’, ‘brighten’, as well as the exposure, the contrast, the brightness, as well as ‘gamma brightness’ are changed.

Pattern extraction was done manually using the open‐source graphics editing program ‘PhotoScape’ (version 3.7, MOOII Tech, Korea). All images have been transformed into grey scale. The largest possible standard elliptical area, which has a standard aspect of ratio, area of each tail was cropped from the images, focusing always on the base of the tail (Figures 2a and 3a).

**FIGURE 4:**
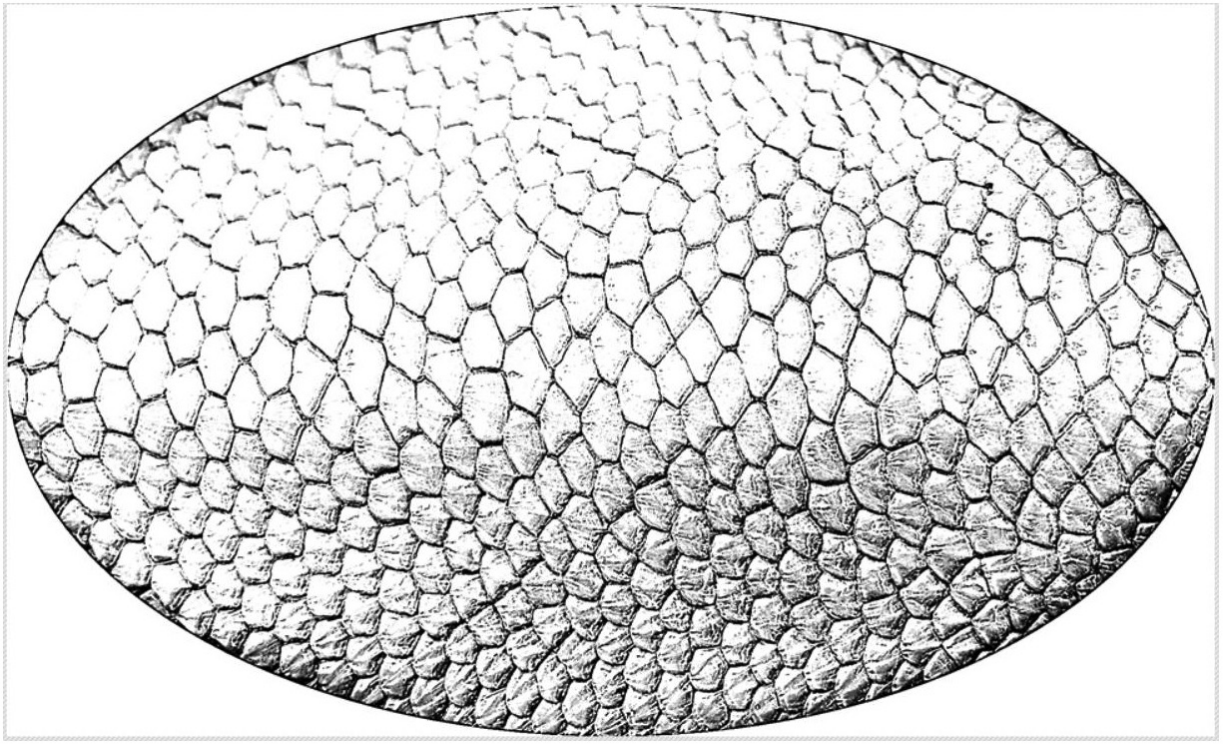
Edited image with an extracted scale pattern of a beaver tail used in the SIFT algorithm.

To emphasise the scale pattern from the images and to correct exposure and standardise all images, the following adjustments of the following photograph parameters have been done. The criteria for image processing were that the images were processed until a line pattern of the scale could be visually. Consequently, all images were qualitatively processed until they were visually similar. The sharpness was increased to 150% for images from both groups (Figures 2c and 3c). Subsequently, the range of ‘intensify’ and ‘brightening’ was increased from 0% to the maximum of 100% for images of both groups (Figures 2d and 3d). The exposure was also increased from 1.00 to the maximum value (5.00) in images from both groups. Likewise, the contrast was increased from 0 to the maximum value of 100 in both groups. The main difference between the images of the groups ‘close’ to the images of the group ‘far’ to obtain the same quality of the images are that images from group ‘close’ had to be processed at the backlight function with ±150% (Figure 2b), the ‘gamma brightness’ had to be increased from 1.00 to values between 2.20 and 2.80 (depending on the respective image) and a reduce in the lightness from 0 to values between −35 and −45. In comparison with that, in images of the group ‘far’ the backlight function with ±200% was used (Figure 3b), the ‘gamma brightness’ had to be increased from 1.00 to values between 3.00 and 3.80, and the brightness was reduced from 0 to values between −10 and −20. The edited images were saved as jpg files (Figure 4).

### 2.3 Pattern detection

We used the SIFT algorithm to extract the patterns and find the matching individuals (Alhwarin et al., 2008; Bolger et al., 2012; Lowe, 1999, 2004b). This algorithm finds and extracts features invariant to scaling, rotation and illumination. A python script for the SIFT algorithm was written and inserted into ‘Visual Studio Code’ (version 1.70.2, Microsoft) on a ‘HP 255 G6 Notebook’ in the ‘Windows 10 Pro’ environment. To execute the mentioned algorithm, four main steps are involved in the SIFT, which are briefly described in the following sections.

1 *Scale‐space extrema detection*: Our grey‐scaled images are searched for the detection of potential keypoints. To reduce the noise in each image, a Gaussian blur technique was used. For this purpose, our images were scaled to an image dimension of 640 × 480 pixels (original 1330 × 889 pixels) with a number of octaves = 4 and a number of scale levels = 5 (Lowe, 2004b). To enhance the features of each image a Difference of Gaussians (DoG) is used, which builds a DoG pyramid with a Gaussian kernel of *σ* = 1.6 and *k* = √2 (Alhwarin et al., 2008; Lowe, 2004b). Once a DoG is found, the local extrema (maxima or minima) are detected by comparing one pixel with its eight neighbours in the same scale, as well as with nine pixels in the next (above) and nine pixels in the previous (below) scales, hence a comparison in total with 26 neighbours in the scale‐space. If it is a local extrema, it is a keypoint, which means it is best represented in this scale (Alhwarin et al., 2008).
2 *Keypoint detection*: These detected keypoints may not be robust to noise and therefore have to be refined. Therefore, keypoints with a low contrast were eliminated. For this purpose, a second order Taylor expansion was computed for each keypoint (Alhwarin et al., 2008; Bolger et al., 2012; Lowe, 2004b). A keypoint is rejected when the resulting contrast threshold value is <0.03 (in magnitude) (Lowe, 2004b). In addition, DoG has a higher response for edges, therefore edges also had to be removed. This was done via a Harris corner detector in a 2 × 2 Hessian matrix. The ratio threshold for the edge is given with *r* = 10 so any keypoint with *r* > 10 was rejected (Lowe, 2004b). Consequently, all low contrast and edge keypoints were discarded and only keypoints of high interest remained.
3 *Orientation assignment*: Because up to this point there was a set of stable keypoints, an orientation is assigned to each keypoint to achieve invariant to image rotation. For this reason, the neighbourhood around a keypoint was taken and the magnitude and orientation was calculated as follows:
$$m\;\left(x,y\right)=\sqrt{{\left(L\left(x+1,y\right)-L\left(x-1,y\right)\right)}^{2}+{\left(L\left(x,y+1\right)-L\left(x,y-1\right)\right)}^{2}}$$
$$\theta \;\left(x,y\right)=\mathrm{ta}n^{-1}\;\left(\left(L\left(x,y+1\right)-L\left(x,y-1\right)\right)/\left(L\left(x+1,y\right)-L\left(x-1,y\right)\right)\right)$$
  (Alhwarin et al., 2008; Lowe, 2004b). After calculating the magnitude and orientation, an orientation histogram was created. This histogram consists of 36 bins covering 360 degrees and was weighted by the magnitude and gaussian‐weighted circular window with σ equal to 1.5 times the scale of a keypoint. The highest peak in the histogram and additionally any peak with an amplitude >80% was used to create a keypoint with an orientation (Alhwarin et al., 2008; Lowe, 2004b). The orientation assignment contributes to a variety of keypoints with the same locations and scale, but with different directions (Alhwarin et al., 2008).
4 *Keypoint descriptor*: The steps above produced a stable set of keypoints that were invariant to scale and rotation. The next step was to generate a descriptor, which is a unique fingerprint to a keypoint, using the neighbouring pixels with their orientation and magnitude. For this a 16 × 16 neighbourhood around a keypoint was taken (Alhwarin et al., 2008; Lowe, 2004b). This neighbourhood was further divided into 16 sub‐blocks of 4 × 4 box size. Furthermore, for every sub‐block a eight‐bin histogram was created, leading to a total of 128 bin values. This was then represented as a vector for forming a keypoint descriptor.

### 2.4 SIFT features matching

**FIGURE 5:**
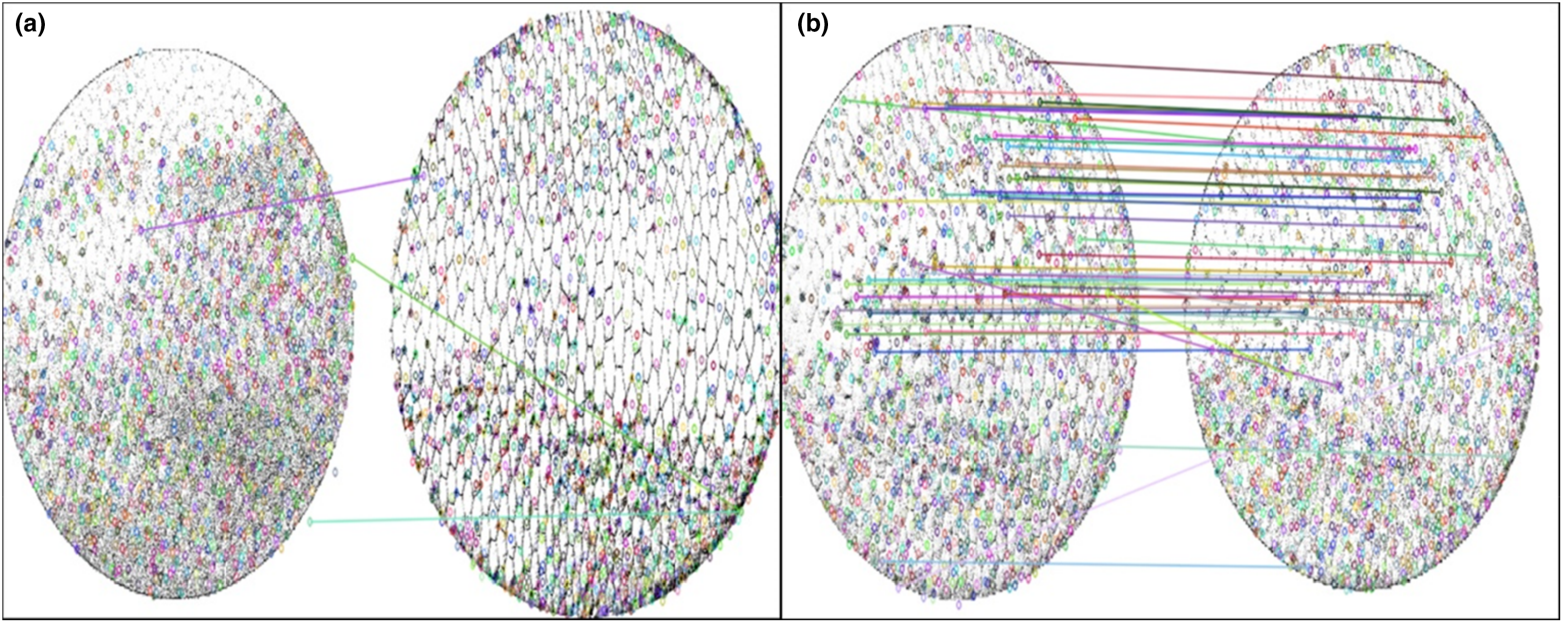
Visualisation of the match between images. The coloured lines indicate the localisation of matching Scale Invariant Features Transform features identified by the algorithm. (a) Visualised the match between two images of two distinct beavers, finding a low amount of matched features (*n* = 3). (b) visualised the match between two images of the same beavers, finding a high amount of matched features (*n* = 54).

In this step the created SIFT features were used for matching (Alhwarin et al., 2008). We used FLANN (Fast Library for Approximate Nearest Neighbours) to match the features. FLANN uses the nearest neighbours approach and runs usually faster than BruceForceMatcher and is particularly well suited to data sets (Muja & Lowe, 2009). The function was used to perform a *k*‐nearest neighbour search with *k* = 2, meaning to find the two nearest neighbours. The found matches were then filtered using a distance ratio test with a threshold value of 0.75 to decide whether to include a match or not (Figure 5).

### 2.5 Training and test data

The data set of 800 images was randomly split into 70% for the database (*n* = 560 images) and 30% for test data (*n* = 240 images). Due to the split of the images into two groups (‘close’ and ‘far’), each group consisted of 280 images for the database and 60 images for test data. Accuracy was calculated by the number of correct predictions divided by the total number of predictions.

## 3 RESULTS

The SIFT algorithm generated a total of 493 matches of which 472 were correct, leading to an accuracy of 95.7% for correctly identifying individual beavers. Confusion matrices were generated for each sample of each group to further investigate the matches (Table 1). In the group ‘close’, 243 predictions were made, of which 238 were correct; resulting in an accuracy of 97.9% (sample 1: 98.4%; sample 2: 95.1%; sample 3: 96.7% and sample 4: 98.4%). The group ‘far’ was able to achieve an accuracy of 93.6% with 234 correct predictions out of 250 total predictions (sample 1: 93.4%; sample 2: 86.9%; sample 3: 95.5% and sample 4: 98.4%).

**TABLE 1:** Confusion matrix for sample 1 of the group ‘close’, representing the beavers 1–25.

	Actual beaver																										
		1	2	3	4	5	6	7	8	9	10	11	12	13	14	15	16	17	18	19	20	21	22	23	24	25	Correct classification (%)
Predicted beaver	1	**1**																1									50.0
	2		**1**																								100.0
	3			**2**																							100.0
	4				**3**																						100.0
	5					**2**																					100.0
	6						**3**																				100.0
	7							**3**																			100.0
	8								**2**																		100.0
	9									**3**																	100.0
	10										**3**																100.0
	11											**3**															100.0
	12												**2**														100.0
	13													**2**													100.0
	14														**3**												100.0
	15															**2**											100.0
	16																**2**										100.0
	17																	**2**									100.0
	18																		**3**								100.0
	19																			**2**							100.0
	20																				**3**						100.0
	21																					**2**					100.0
	22																						**3**				100.0
	23																							**3**			100.0
	24																								**2**		100.0
	25																									**2**	100.0
	Mean correct																										98.0

*Note*: The rows show the actual animals, the columns the predicted ones. Numbers reflect the counts of the associated images per individual. Numbers in bold and highlighted in blue represent correct predictions, numbers without a background represent incorrect predictions.

## 4 DISCUSSION

Using a SIFT algorithm, we have shown that it is possible to distinguish beavers individually by the pattern of scales on the dorsal sides of their tails. Moreover, we were able to prove that the tail pattern of the beaver tail was unique for most individuals and was therefore suitable as a distinguishing feature (Hinds et al., 2023). We achieved very satisfactory results for the two groups ‘close’ and ‘far’ with accuracies of >90%. When distinguishing beavers individually based on natural characteristics through images, the decisive point is that the characteristics to be distinguished can also be clearly recognised in the images. It is known that image quality also influences the matching in other computer‐matching programs (Lahiri et al., 2011). The probability of correct matching increases significantly when the low‐quality images are excluded (Whitehead, 1990). Thus, the selection of suitable images and the clean elaboration of the pattern during image processing is an important factor.

Comparing our computer‐assisted analysis with other studies showed that these applications were useful in the analysis of images from the field. The SIFT algorithm used in the software ‘Wild‐ID’ was also successful in the individual recognition of 600 giraffes by comparing 1026 images taken by a Digital Single‐Lens Reflex (DSLR) camera in the field (Bolger et al., 2012). Also, a study to recognise elephants (*Loxodonta* spp.) by the shape of the nicks on their ears using human‐made images from the field showed successful results using a semi‐automated computer approach (Ardovini et al., 2007). Also, zebras (*Equus grevyi* and *E*. *quagga*) can be successfully individually identified using SLR recordings in the field using an algorithm (StripeCode) (Lahiri et al., 2011). Even in a species (the brown bear, *U. arctos*) that has no obvious pigmentation patterns, individuals could be differentiated from DSLR and camera trap images of their facial features using a CNN algorithm (BearID) (Clapham et al., 2020).

Moreover, the use of camera traps in combination with computer‐aided detection systems is an interesting topic. However, only the differentiation of different species has been tested so far (Norouzzadeh et al., 2018). It was shown that the software ‘ClassifyMe’ enables the automated identification of animal species, that is cats (Felidae), dogs (Canidae), foxes (Vulpini) and macropods (Macropodidae) using camera trap images (Falzon et al., 2019). The results were very satisfactory both with natural illumination and with infrared illumination with accuracies of >90% and in addition, promising for the possible further use of individual differentiation. Furthermore, it was shown that modifications of camera traps by an external lens enhance the quality of beaver tail images (Dytkowicz et al., 2023). The use of this additional external lens enables the image sharpness to be improved and thus a clearer visualisation of the pattern structure. Additionally, the external lens increases the number of good images by a factor of four and can therefore, most likely, be used for individual identification. This appears to be a promising new approach of a non‐invasive method for the individual identification of beavers in the future.

Our method could be used as a non‐invasive tool for ecologists and wildlife managers to obtain an overview of the number of beavers and to determine their distribution pattern. This study revealed satisfactory results for beaver tail images of deceased beavers taken under good light conditions and gives reason for further investigations. Therefore, the next step should be to take images of tails in the field with camera traps and evaluate them by using the SIFT algorithm to find out if it is possible to identify different beavers. Since it has been shown that the quality of the images was a decisive factor for success, the recordings using modified camera traps with the help of an additional lens should be used (Dytkowicz et al., 2023).

## AUTHOR CONTRIBUTIONS

**Margarete Dytkowicz:** Conceptualization (equal); data curation (equal); investigation (equal); methodology (equal); project administration (lead); validation (equal); writing – original draft (lead); writing – review and editing (lead). **Marcello Tania:** Conceptualization (equal); data curation (equal); formal analysis (lead); investigation (equal); methodology (lead); software (lead); validation (equal); writing – original draft (equal); writing – review and editing (supporting). **Rachel Hinds:** Conceptualization (equal); data curation (equal); investigation (equal); methodology (equal); resources (lead); validation (equal); writing – original draft (equal); writing – review and editing (supporting). **William M. Megill:** Conceptualization (supporting); investigation (equal); methodology (equal); supervision (lead); writing – original draft (supporting); writing – review and editing (supporting). **Tillmann K. Buttschardt:** Conceptualization (supporting); investigation (equal); supervision (lead); writing – original draft (supporting); writing – review and editing (supporting). **Frank Rosell:** Conceptualization (equal); data curation (equal); investigation (equal); methodology (equal); resources (lead); validation (equal); writing – original draft (supporting); writing – review and editing (supporting).

## CONFLICT OF INTEREST STATEMENT

No competing interests.

## Data Availability

Visual Studio Code, version 1.70.2, Microsoft, is an open‐source code editor available at https://code.visualstudio.com/docs/?dv=win. The open‐source image editing program PhotoScape, version 3.7, MOOII Tech, Korea, is available at https://photoscape.de.softonic.com/. BeaverID is an open‐source application available on GitHub at https://github.com/MaDyt2508/beaverid. The code is also archived at https://doi.org/10.5281/zenodo.8311347. Raw and edited images will only be available upon request to the author.
